# [(*E*)-*N*′-(5-Chloro-2-oxidobenzyl­idene-κ*O*)-3,4,5-trimeth­oxy­benzohydrazidato-κ^2^ *N*′,*O*](pyridine-κ*N*)copper(II)

**DOI:** 10.1107/S1600536811009901

**Published:** 2011-04-07

**Authors:** Yu-Min Wang, Xiao-Hui Lin, Zhen Chen, Hong-Li Jiang, Chang-Jun Zhang

**Affiliations:** a School of Chemistry and Chemical Engineering, Taishan Medical University, Tai an 271016, People’s Republic of China

## Abstract

In the title compound, [Cu(C_17_H_15_ClN_2_O_5_)(C_5_H_5_N)], the Cu^II^ atom is coordinated by one N atom and two O atoms from an anionic salicyl­aldehyde benzoyl­hydrazone ligand and one pyridine N atom in a distorted square-planar geometry. The bonds displays the usual elongation with mean Cu—O and Cu—N bond lengths of 1.926 and 1.976 Å, respectively. The pyridine ring makes dihedral angles of 26.12 (13) and 11.08 (12)°, respectively, with the trimeth­oxy­phenyl and phenolate rings, which make a dihedral angle of 16.05 (12)° with one another.

## Related literature

For the biolgical activity of salicyl­aldehyde derivatives, see: Chan *et al.* (1995 ▶); Ranford *et al.* (1998 ▶); Monfared *et al.* (2009 ▶). For related structures, see: Lee *et al.* (2003 ▶).
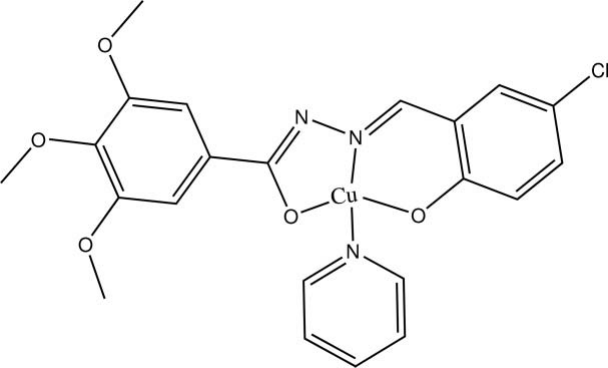


## Experimental

### 

#### Crystal data

[Cu(C_17_H_15_ClN_2_O_5_)(C_5_H_5_N)]
*M* *_r_* = 505.40
Monoclinic, 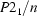

*a* = 14.274 (4) Å
*b* = 7.5763 (18) Å
*c* = 20.753 (5) Å
β = 99.108 (4)°
*V* = 2216.1 (9) Å^3^
*Z* = 4
Mo *K*α radiation
μ = 1.15 mm^−1^
*T* = 298 K
0.19 × 0.16 × 0.12 mm

#### Data collection

Bruker SMART CCD area-detector diffractometer
Absorption correction: multi-scan (*SADABS*; Bruker, 1998 ▶) *T*_min_ = 0.812, *T*_max_ = 0.875
11265 measured reflections
3908 independent reflections
3184 reflections with *I* > 2σ(*I*)
*R*_int_ = 0.027

#### Refinement

*R*[*F* ^2^ > 2σ(*F* ^2^)] = 0.031
*wR*(*F* ^2^) = 0.086
*S* = 1.04
3908 reflections
289 parameters
H-atom parameters constrained
Δρ_max_ = 0.27 e Å^−3^
Δρ_min_ = −0.37 e Å^−3^

### 

Data collection: *SMART* (Bruker, 1998 ▶); cell refinement: *SAINT* (Bruker, 1998 ▶); data reduction: *SAINT*; program(s) used to solve structure: *SHELXS97* (Sheldrick, 2008 ▶); program(s) used to refine structure: *SHELXL97* (Sheldrick, 2008 ▶); molecular graphics: *SHELXTL* (Sheldrick, 2008 ▶); software used to prepare material for publication: *SHELXTL*.

## Supplementary Material

Crystal structure: contains datablocks I, global. DOI: 10.1107/S1600536811009901/jh2271sup1.cif

Structure factors: contains datablocks I. DOI: 10.1107/S1600536811009901/jh2271Isup2.hkl

Additional supplementary materials:  crystallographic information; 3D view; checkCIF report

## Figures and Tables

**Fig. 1.**
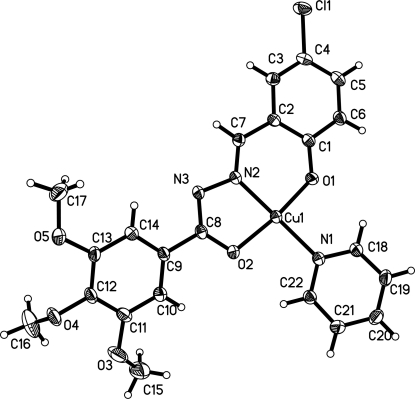
The molecular structure of (I), showing displacement ellipsoids drawn at the 50% probability level.

**Fig. 2.**
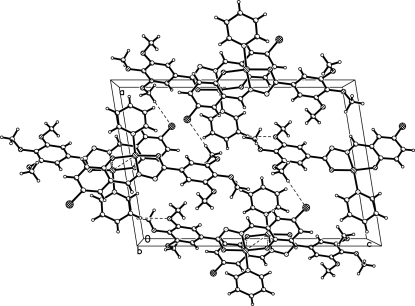
Packing diagram.

## supplementary crystallographic information

### Comment

Transition metal complexes with potential biological activity are the focus of extensive investigation. Salicylaldehyde benzoylhydrazone possess mild bacteriostatic activity and inhibits DNA synthesis and cell growth (Chan *et al.*; 1995). Salicylaldehyde acetylhydrazone displays radioprotective properties (Ranford *et al.*; 1998). Because of the biological interest in this type of chelate system, several structural studies have been carried out on copper with their analogues (Lee *et al.*; 2003). The copper(II) complex was shown to be significantly more potent than the metal-free chelate, leading to the suggestion that the metal complex was the biologically active species (Monfared *et al.*; 2009). We report here the crystal structure of the title compound, (I) (Fig. 1). It can be seen that the coordination environment of the copper atom consists of two oxygen atoms and one nitrogen atom from the salicylaldehyde benzoylhydrazone, and one nitrogen atom from the pyridine groups, making up a distorted square-planar environment. The bond length displays the usual elongation: Cu—O = 1.9256 (average) and Cu—N = 1.9755 (average). The pyridine ring makes dihedral angles of 26.12° and 11.08°, respectively, with the C9—C14 and C1—C6 phenyl rings. The C1—C6 benzene ring system makes a dihedral angle of 16.05° with the other C9—C14 benzene ring.

### Experimental

Mixture of 20 ml aqueous solution of copper (II) acetate (0.2 mmol) with 2 ml of pyridine was stirred with 20 ml e thanolic solution of (*E*)-*N*'-(5-chloro-2-hydroxybenzylidene) -3,4,5-trimethoxybenzohydrazide for 1 h. The resulted solution was leaved in dark place for evaporation. After 1 week of stating blue needle-like shape crystals were grown.

### Refinement

All H atoms were placed in geometrically calculated positions and refined using a riding model with C—H = 0.97 Å (for CH_2_ groups) and 0.96 Å (for CH_3_ groups), their isotropic displacement parameters were set to 1.2 times (1.5 times for CH_3_ groups) the equivalent displacement parameter of their parent atoms.

### Crystal data

**Table:**

[Cu(C_17_H_15_ClN_2_O_5_)(C_5_H_5_N)]	*F*(000) = 1036
*M**_r_* = 505.40	*D*_x_ = 1.515 Mg m^−^^3^
Monoclinic, *P*2_1_/*n*	Mo *K*α radiation, λ = 0.71073 Å
Hall symbol: -P 2yn	Cell parameters from 3527 reflections
*a* = 14.274 (4) Å	θ = 2.9–25.3°
*b* = 7.5763 (18) Å	µ = 1.15 mm^−^^1^
*c* = 20.753 (5) Å	*T* = 298 K
β = 99.108 (4)°	Block, blue
*V* = 2216.1 (9) Å^3^	0.19 × 0.16 × 0.12 mm
*Z* = 4	

### Data collection

**Table:**

Bruker SMART CCD area-detector diffractometer	3908 independent reflections
Radiation source: fine-focus sealed tube	3184 reflections with *I* > 2σ(*I*)
graphite	*R*_int_ = 0.027
φ and ω scans	θ_max_ = 25.0°, θ_min_ = 1.6°
Absorption correction: multi-scan (*SADABS*; Bruker, 1998)	*h* = −15→17
*T*_min_ = 0.812, *T*_max_ = 0.875	*k* = −8→9
11265 measured reflections	*l* = −20→24

### Refinement

**Table:**

Refinement on *F*^2^	Primary atom site location: structure-invariant direct methods
Least-squares matrix: full	Secondary atom site location: difference Fourier map
*R*[*F*^2^ > 2σ(*F*^2^)] = 0.031	Hydrogen site location: inferred from neighbouring sites
*wR*(*F*^2^) = 0.086	H-atom parameters constrained
*S* = 1.04	*w* = 1/[σ^2^(*F*_o_^2^) + (0.0423*P*)^2^ + 0.808*P*] where *P* = (*F*_o_^2^ + 2*F*_c_^2^)/3
3908 reflections	(Δ/σ)_max_ < 0.001
289 parameters	Δρ_max_ = 0.27 e Å^−^^3^
0 restraints	Δρ_min_ = −0.37 e Å^−^^3^

### Special details

**Table:**

Geometry. All e.s.d.'s (except the e.s.d. in the dihedral angle between two l.s. planes) are estimated using the full covariance matrix. The cell e.s.d.'s are taken into account individually in the estimation of e.s.d.'s in distances, angles and torsion angles; correlations between e.s.d.'s in cell parameters are only used when they are defined by crystal symmetry. An approximate (isotropic) treatment of cell e.s.d.'s is used for estimating e.s.d.'s involving l.s. planes.
Refinement. Refinement of *F*^2^ against ALL reflections. The weighted *R*-factor *wR* and goodness of fit *S* are based on *F*^2^, conventional *R*-factors *R* are based on *F*, with *F* set to zero for negative *F*^2^. The threshold expression of *F*^2^ > σ(*F*^2^) is used only for calculating *R*-factors(gt) *etc*. and is not relevant to the choice of reflections for refinement. *R*-factors based on *F*^2^ are statistically about twice as large as those based on *F*, and *R*- factors based on ALL data will be even larger.

### Fractional atomic coordinates and isotropic or equivalent isotropic displacement parameters (Å^2^)

**Table:**

	*x*	*y*	*z*	*U*_iso_*/*U*_eq_	
Cu1	0.54442 (2)	0.14856 (4)	0.056192 (13)	0.03244 (11)	
Cl1	0.26557 (6)	0.62229 (10)	−0.21238 (3)	0.0569 (2)	
O1	0.52842 (12)	0.1751 (2)	−0.03651 (8)	0.0393 (4)	
O2	0.54254 (12)	0.1224 (2)	0.14894 (8)	0.0364 (4)	
O3	0.5244 (2)	−0.0473 (3)	0.38634 (10)	0.0825 (8)	
O4	0.41727 (15)	0.2050 (3)	0.42842 (8)	0.0565 (5)	
O5	0.33613 (15)	0.4566 (3)	0.34986 (9)	0.0655 (6)	
N1	0.67522 (14)	0.0438 (3)	0.05888 (9)	0.0324 (4)	
N2	0.43295 (14)	0.2875 (3)	0.06254 (9)	0.0312 (4)	
N3	0.40808 (14)	0.2968 (3)	0.12482 (9)	0.0352 (5)	
C1	0.46948 (17)	0.2826 (3)	−0.07312 (11)	0.0337 (5)	
C2	0.39692 (17)	0.3819 (3)	−0.04970 (11)	0.0315 (5)	
C3	0.33515 (18)	0.4867 (3)	−0.09362 (12)	0.0370 (6)	
H3	0.2878	0.5516	−0.0784	0.044*	
C4	0.34372 (18)	0.4943 (3)	−0.15816 (12)	0.0386 (6)	
C5	0.4148 (2)	0.3997 (3)	−0.18158 (12)	0.0421 (6)	
H5	0.4209	0.4063	−0.2255	0.051*	
C6	0.47585 (19)	0.2969 (3)	−0.13977 (12)	0.0399 (6)	
H6	0.5231	0.2346	−0.1561	0.048*	
C7	0.38229 (18)	0.3777 (3)	0.01742 (12)	0.0346 (6)	
H7	0.3329	0.4448	0.0289	0.042*	
C8	0.47163 (17)	0.2079 (3)	0.16524 (11)	0.0328 (5)	
C9	0.45815 (17)	0.2063 (3)	0.23496 (11)	0.0333 (5)	
C10	0.50005 (19)	0.0761 (4)	0.27666 (12)	0.0423 (6)	
H10	0.5382	−0.0089	0.2615	0.051*	
C11	0.4850 (2)	0.0729 (4)	0.34094 (12)	0.0464 (7)	
C12	0.42910 (18)	0.2013 (4)	0.36393 (11)	0.0411 (6)	
C13	0.38825 (18)	0.3340 (4)	0.32232 (12)	0.0410 (6)	
C14	0.40278 (18)	0.3369 (3)	0.25749 (12)	0.0393 (6)	
H14	0.3757	0.4253	0.2295	0.047*	
C15	0.5491 (3)	−0.2135 (5)	0.36663 (18)	0.0856 (12)	
H15A	0.4979	−0.2609	0.3360	0.128*	
H15B	0.5616	−0.2899	0.4039	0.128*	
H15C	0.6049	−0.2046	0.3464	0.128*	
C16	0.3549 (3)	0.0777 (6)	0.44687 (19)	0.1005 (15)	
H16A	0.2959	0.0819	0.4174	0.151*	
H16B	0.3437	0.1018	0.4904	0.151*	
H16C	0.3826	−0.0374	0.4454	0.151*	
C17	0.2831 (3)	0.5821 (5)	0.30812 (17)	0.0887 (13)	
H17A	0.3257	0.6550	0.2883	0.133*	
H17B	0.2469	0.6545	0.3331	0.133*	
H17C	0.2409	0.5216	0.2747	0.133*	
C18	0.71659 (18)	0.0346 (4)	0.00516 (12)	0.0414 (6)	
H18	0.6856	0.0849	−0.0332	0.050*	
C19	0.80282 (19)	−0.0460 (4)	0.00440 (13)	0.0497 (7)	
H19	0.8296	−0.0487	−0.0336	0.060*	
C20	0.8487 (2)	−0.1221 (4)	0.06053 (14)	0.0481 (7)	
H20	0.9064	−0.1793	0.0610	0.058*	
C21	0.80784 (19)	−0.1126 (4)	0.11634 (13)	0.0447 (7)	
H21	0.8378	−0.1629	0.1551	0.054*	
C22	0.72216 (18)	−0.0276 (3)	0.11392 (12)	0.0368 (6)	
H22	0.6957	−0.0192	0.1520	0.044*	

### Atomic displacement parameters (Å^2^)

**Table:**

	*U*^11^	*U*^22^	*U*^33^	*U*^12^	*U*^13^	*U*^23^
Cu1	0.03527 (18)	0.03684 (19)	0.02615 (17)	0.00328 (13)	0.00780 (12)	0.00003 (12)
Cl1	0.0675 (5)	0.0611 (5)	0.0370 (4)	0.0104 (4)	−0.0071 (3)	0.0082 (3)
O1	0.0453 (11)	0.0446 (10)	0.0289 (9)	0.0119 (8)	0.0083 (8)	0.0031 (8)
O2	0.0374 (10)	0.0434 (10)	0.0296 (9)	0.0054 (8)	0.0092 (7)	−0.0010 (7)
O3	0.136 (2)	0.0742 (16)	0.0376 (12)	0.0460 (16)	0.0132 (13)	0.0155 (11)
O4	0.0654 (13)	0.0796 (14)	0.0270 (10)	−0.0020 (11)	0.0151 (9)	0.0004 (9)
O5	0.0726 (15)	0.0912 (16)	0.0354 (11)	0.0362 (13)	0.0167 (10)	−0.0040 (11)
N1	0.0345 (11)	0.0330 (11)	0.0300 (11)	−0.0024 (9)	0.0065 (9)	−0.0012 (9)
N2	0.0341 (11)	0.0341 (11)	0.0261 (10)	−0.0016 (9)	0.0071 (8)	0.0000 (9)
N3	0.0374 (12)	0.0442 (12)	0.0253 (10)	0.0027 (10)	0.0088 (9)	0.0001 (9)
C1	0.0393 (14)	0.0309 (13)	0.0310 (13)	−0.0036 (11)	0.0061 (10)	−0.0003 (11)
C2	0.0351 (13)	0.0283 (13)	0.0308 (13)	−0.0037 (10)	0.0044 (10)	0.0007 (10)
C3	0.0400 (15)	0.0337 (13)	0.0368 (14)	0.0004 (11)	0.0043 (11)	0.0005 (11)
C4	0.0484 (16)	0.0319 (13)	0.0321 (14)	−0.0032 (12)	−0.0038 (11)	0.0046 (11)
C5	0.0558 (17)	0.0419 (15)	0.0285 (13)	−0.0072 (13)	0.0063 (12)	0.0020 (11)
C6	0.0484 (16)	0.0393 (14)	0.0339 (14)	0.0017 (12)	0.0125 (12)	−0.0002 (11)
C7	0.0367 (14)	0.0347 (13)	0.0333 (13)	0.0020 (11)	0.0083 (11)	−0.0010 (11)
C8	0.0365 (14)	0.0342 (13)	0.0285 (12)	−0.0069 (11)	0.0076 (11)	−0.0035 (10)
C9	0.0328 (13)	0.0422 (14)	0.0255 (12)	−0.0041 (11)	0.0064 (10)	−0.0023 (11)
C10	0.0471 (16)	0.0467 (15)	0.0342 (14)	0.0078 (13)	0.0097 (12)	−0.0018 (12)
C11	0.0548 (18)	0.0527 (17)	0.0309 (14)	0.0034 (14)	0.0043 (12)	0.0055 (12)
C12	0.0426 (15)	0.0576 (17)	0.0242 (13)	−0.0049 (13)	0.0083 (11)	−0.0043 (12)
C13	0.0369 (14)	0.0573 (17)	0.0300 (13)	0.0056 (12)	0.0094 (11)	−0.0066 (12)
C14	0.0403 (15)	0.0478 (15)	0.0297 (13)	0.0050 (12)	0.0053 (11)	−0.0002 (11)
C15	0.110 (3)	0.072 (2)	0.075 (2)	0.030 (2)	0.018 (2)	0.024 (2)
C16	0.132 (4)	0.113 (3)	0.069 (3)	−0.036 (3)	0.056 (3)	0.000 (2)
C17	0.089 (3)	0.115 (3)	0.062 (2)	0.061 (3)	0.009 (2)	−0.010 (2)
C18	0.0392 (15)	0.0530 (16)	0.0325 (14)	0.0007 (12)	0.0073 (11)	0.0036 (12)
C19	0.0411 (16)	0.071 (2)	0.0405 (16)	0.0039 (14)	0.0158 (12)	−0.0036 (14)
C20	0.0358 (15)	0.0576 (18)	0.0512 (17)	0.0086 (13)	0.0082 (13)	−0.0046 (14)
C21	0.0419 (16)	0.0521 (17)	0.0387 (15)	0.0044 (13)	0.0016 (12)	0.0022 (12)
C22	0.0392 (14)	0.0402 (14)	0.0313 (13)	−0.0005 (11)	0.0065 (11)	−0.0015 (11)

### Geometric parameters (Å, °)

**Table:**

Cu1—O1	1.9119 (16)	C7—H7	0.9300
Cu1—N2	1.930 (2)	C8—C9	1.490 (3)
Cu1—O2	1.9394 (16)	C9—C10	1.384 (4)
Cu1—N1	2.021 (2)	C9—C14	1.393 (3)
Cl1—C4	1.747 (3)	C10—C11	1.385 (3)
O1—C1	1.321 (3)	C10—H10	0.9300
O2—C8	1.291 (3)	C11—C12	1.390 (4)
O3—C11	1.366 (3)	C12—C13	1.392 (4)
O3—C15	1.386 (4)	C13—C14	1.394 (3)
O4—C12	1.375 (3)	C14—H14	0.9300
O4—C16	1.406 (4)	C15—H15A	0.9600
O5—C13	1.370 (3)	C15—H15B	0.9600
O5—C17	1.421 (4)	C15—H15C	0.9600
N1—C22	1.343 (3)	C16—H16A	0.9600
N1—C18	1.344 (3)	C16—H16B	0.9600
N2—C7	1.285 (3)	C16—H16C	0.9600
N2—N3	1.395 (2)	C17—H17A	0.9600
N3—C8	1.319 (3)	C17—H17B	0.9600
C1—C6	1.405 (3)	C17—H17C	0.9600
C1—C2	1.426 (3)	C18—C19	1.376 (4)
C2—C3	1.408 (3)	C18—H18	0.9300
C2—C7	1.441 (3)	C19—C20	1.370 (4)
C3—C4	1.365 (3)	C19—H19	0.9300
C3—H3	0.9300	C20—C21	1.378 (4)
C4—C5	1.391 (4)	C20—H20	0.9300
C5—C6	1.371 (4)	C21—C22	1.376 (4)
C5—H5	0.9300	C21—H21	0.9300
C6—H6	0.9300	C22—H22	0.9300
			
O1—Cu1—N2	92.45 (7)	C11—C10—H10	120.1
O1—Cu1—O2	172.44 (7)	O3—C11—C10	124.5 (3)
N2—Cu1—O2	81.20 (7)	O3—C11—C12	115.2 (2)
O1—Cu1—N1	91.82 (7)	C10—C11—C12	120.2 (2)
N2—Cu1—N1	168.60 (8)	O4—C12—C11	120.9 (2)
O2—Cu1—N1	95.18 (7)	O4—C12—C13	119.0 (2)
C1—O1—Cu1	127.39 (15)	C11—C12—C13	120.0 (2)
C8—O2—Cu1	110.09 (14)	O5—C13—C12	115.6 (2)
C11—O3—C15	119.9 (2)	O5—C13—C14	124.4 (2)
C12—O4—C16	115.6 (2)	C12—C13—C14	119.9 (2)
C13—O5—C17	118.1 (2)	C9—C14—C13	119.5 (2)
C22—N1—C18	117.3 (2)	C9—C14—H14	120.3
C22—N1—Cu1	121.05 (16)	C13—C14—H14	120.3
C18—N1—Cu1	121.52 (17)	O3—C15—H15A	109.5
C7—N2—N3	116.9 (2)	O3—C15—H15B	109.5
C7—N2—Cu1	127.97 (16)	H15A—C15—H15B	109.5
N3—N2—Cu1	115.09 (14)	O3—C15—H15C	109.5
C8—N3—N2	108.26 (19)	H15A—C15—H15C	109.5
O1—C1—C6	118.6 (2)	H15B—C15—H15C	109.5
O1—C1—C2	124.0 (2)	O4—C16—H16A	109.5
C6—C1—C2	117.3 (2)	O4—C16—H16B	109.5
C3—C2—C1	119.4 (2)	H16A—C16—H16B	109.5
C3—C2—C7	117.8 (2)	O4—C16—H16C	109.5
C1—C2—C7	122.8 (2)	H16A—C16—H16C	109.5
C4—C3—C2	121.0 (2)	H16B—C16—H16C	109.5
C4—C3—H3	119.5	O5—C17—H17A	109.5
C2—C3—H3	119.5	O5—C17—H17B	109.5
C3—C4—C5	120.3 (2)	H17A—C17—H17B	109.5
C3—C4—Cl1	120.5 (2)	O5—C17—H17C	109.5
C5—C4—Cl1	119.17 (19)	H17A—C17—H17C	109.5
C6—C5—C4	119.8 (2)	H17B—C17—H17C	109.5
C6—C5—H5	120.1	N1—C18—C19	122.9 (2)
C4—C5—H5	120.1	N1—C18—H18	118.5
C5—C6—C1	122.2 (2)	C19—C18—H18	118.5
C5—C6—H6	118.9	C20—C19—C18	119.0 (2)
C1—C6—H6	118.9	C20—C19—H19	120.5
N2—C7—C2	124.4 (2)	C18—C19—H19	120.5
N2—C7—H7	117.8	C19—C20—C21	119.0 (3)
C2—C7—H7	117.8	C19—C20—H20	120.5
O2—C8—N3	125.3 (2)	C21—C20—H20	120.5
O2—C8—C9	118.5 (2)	C22—C21—C20	119.0 (2)
N3—C8—C9	116.1 (2)	C22—C21—H21	120.5
C10—C9—C14	120.6 (2)	C20—C21—H21	120.5
C10—C9—C8	120.2 (2)	N1—C22—C21	122.7 (2)
C14—C9—C8	119.2 (2)	N1—C22—H22	118.6
C9—C10—C11	119.8 (2)	C21—C22—H22	118.6
C9—C10—H10	120.1		
			
N2—Cu1—O1—C1	10.7 (2)	Cu1—O2—C8—C9	−179.27 (16)
N1—Cu1—O1—C1	−158.7 (2)	N2—N3—C8—O2	−2.2 (3)
N2—Cu1—O2—C8	−0.24 (15)	N2—N3—C8—C9	178.60 (19)
N1—Cu1—O2—C8	168.87 (15)	O2—C8—C9—C10	−20.0 (3)
O1—Cu1—N1—C22	−171.09 (18)	N3—C8—C9—C10	159.3 (2)
N2—Cu1—N1—C22	76.9 (4)	O2—C8—C9—C14	160.2 (2)
O2—Cu1—N1—C22	6.06 (19)	N3—C8—C9—C14	−20.5 (3)
O1—Cu1—N1—C18	5.5 (2)	C14—C9—C10—C11	1.6 (4)
N2—Cu1—N1—C18	−106.5 (4)	C8—C9—C10—C11	−178.2 (2)
O2—Cu1—N1—C18	−177.36 (19)	C15—O3—C11—C10	−27.1 (5)
O1—Cu1—N2—C7	−8.4 (2)	C15—O3—C11—C12	155.1 (3)
O2—Cu1—N2—C7	175.7 (2)	C9—C10—C11—O3	−178.7 (3)
N1—Cu1—N2—C7	103.5 (4)	C9—C10—C11—C12	−0.9 (4)
O1—Cu1—N2—N3	175.03 (16)	C16—O4—C12—C11	−76.1 (4)
O2—Cu1—N2—N3	−0.85 (15)	C16—O4—C12—C13	107.2 (3)
N1—Cu1—N2—N3	−73.0 (4)	O3—C11—C12—O4	1.2 (4)
C7—N2—N3—C8	−175.3 (2)	C10—C11—C12—O4	−176.8 (2)
Cu1—N2—N3—C8	1.7 (2)	O3—C11—C12—C13	177.8 (3)
Cu1—O1—C1—C6	172.93 (17)	C10—C11—C12—C13	−0.2 (4)
Cu1—O1—C1—C2	−9.2 (3)	C17—O5—C13—C12	−172.3 (3)
O1—C1—C2—C3	−177.2 (2)	C17—O5—C13—C14	8.6 (4)
C6—C1—C2—C3	0.7 (3)	O4—C12—C13—O5	−1.9 (4)
O1—C1—C2—C7	1.6 (4)	C11—C12—C13—O5	−178.6 (3)
C6—C1—C2—C7	179.5 (2)	O4—C12—C13—C14	177.3 (2)
C1—C2—C3—C4	0.1 (4)	C11—C12—C13—C14	0.6 (4)
C7—C2—C3—C4	−178.8 (2)	C10—C9—C14—C13	−1.2 (4)
C2—C3—C4—C5	−0.8 (4)	C8—C9—C14—C13	178.7 (2)
C2—C3—C4—Cl1	179.08 (18)	O5—C13—C14—C9	179.2 (3)
C3—C4—C5—C6	0.7 (4)	C12—C13—C14—C9	0.1 (4)
Cl1—C4—C5—C6	−179.1 (2)	C22—N1—C18—C19	1.0 (4)
C4—C5—C6—C1	0.1 (4)	Cu1—N1—C18—C19	−175.7 (2)
O1—C1—C6—C5	177.2 (2)	N1—C18—C19—C20	0.7 (4)
C2—C1—C6—C5	−0.8 (4)	C18—C19—C20—C21	−1.2 (4)
N3—N2—C7—C2	−179.1 (2)	C19—C20—C21—C22	0.3 (4)
Cu1—N2—C7—C2	4.4 (4)	C18—N1—C22—C21	−2.0 (4)
C3—C2—C7—N2	179.7 (2)	Cu1—N1—C22—C21	174.66 (19)
C1—C2—C7—N2	0.9 (4)	C20—C21—C22—N1	1.5 (4)
Cu1—O2—C8—N3	1.5 (3)		
